# Molecular identification of *Trichinella spiralis* nudix hydrolase and its induced protective immunity against trichinellosis in BALB/c mice

**DOI:** 10.1186/s13071-014-0600-9

**Published:** 2014-12-19

**Authors:** Shao Rong Long, Zhong Quan Wang, Ruo Dan Liu, Li Na Liu, Ling Ge Li, Peng Jiang, Xi Zhang, Zi Fang Zhang, Hai Ning Shi, Jing Cui

**Affiliations:** Department of Parasitology, Medical College of Zhengzhou University, Zhengzhou, 450052 P. R. China; Department of Immunology, Massachusetts General Hospital and Harvard Medical School, Charlestown, MA 02129 USA

**Keywords:** *Trichinella spiralis*, Nudix hydrolases, Protective immunity, Vaccine

## Abstract

**Background:**

Nudix hydrolases (Nd) is a widespread superfamily, which is found in all classes of organism, hydrolyse a wide range of organic pyrophosphates and has a ‘housecleaning’ function. The previous study showed that *Trichinella spiralis* Nd (TsNd) bound to intestinal epithelial cells (IECs), and the vaccination of mice with T7 phage-displayed TsNd polypeptides produced protective immunity. The aim of this study was to clone, express and identify the full-length TsNd and to investigate its immune protection against *T. spiralis* infection.

**Methods:**

The full-length cDNA sequence of TsNd gene encoding a 46 kDa protein from *T. spiralis* intestinal infective larvae (IIL) was cloned and identified. The antigenicity of rTsNd was analyzed by Western blot. Transcription and expression of TsNd at *T. spiralis* different stages were observed by RT-PCR and IFT. The levels of the specific total IgG, IgG1 and IgG2a antibodies to rTsNd were determined by ELISA. The immune protection of rTsNd against *T. spiralis* infection was investigated.

**Results:**

Sequence and phylogenetic analysis revealed that TsNd had a nudix motif located at 226-244aa, which had high homology and the closest evolutionary status with *T. pseudospiralis*. The rTsNd was obtained after expression and purification. Western blot analysis showed that anti-rTsNd serum recognized the native TsNd protein in crude antigens of muscle larvae (ML), IIL, adult worms (AW) and newborn larvae (NBL), and ES antigens of ML. Transcription and expression of TsNd gene was observed in all developmental stages of *T. spiralis* (ML, IIL, AW and NBL), with high level expression in IIL. An immunolocalization analysis identified TsNd in the cuticle, stichocytes and reproductive organs of the parasite. Following immunization, anti-rTsNd IgG levels were increased, and the levels of IgG1 were more significantly higher than that of IgG2a. After a challenge infection with *T. spiralis*, mice immunized with the rTsNd displayed a 57.7% reduction in adult worms and a 56.9% reduction in muscle larval burden.

**Conclusions:**

TsNd induced a partial protective immunity in mice and could be considered as a novel candidate vaccine antigen against trichinellosis.

## Background

Trichinellosis is a serious foodborne parasitic zoonosis caused by eating raw or undercooked meat contaminated with infective larvae of the nematode genus *Trichinella* [1]. *Trichinella* infection has been documented in 66 countries of the world, and is considered as an emerging/re-emerging disease [2,3]. In the past several decades, many outbreaks of human trichinellosis have been reported in different areas of the world [4]. From 2004 to 2009, 15 outbreaks of human trichinellosis, with 1387 cases and 4 deaths, were reported in China [5]. Pork is the most important source of human *Trichinella* infection in China [6]. Trichinellosis is not only a public health hazard but also an economic problem in porcine animal production and food safety [7]. Thus, the development of vaccines capable of preventing swine from becoming infected is a promising approach for control of trichinellosis [8-10].

Nudix hydrolases (Nd) is a widespread superfamily, which is found in all classes of organism, hydrolyse a wide range of organic pyrophosphates and has a ‘housecleaning’ function, that is to eliminate potentially toxic nucleotide metabolites from the cells and to regulate the concentrations of nucleotide cofactors and signalling molecules for optimal cell growth and survival [11,12]. Some Nd have been identified and characterized, and they control a variety of metabolites and are pertinent to a wide range of physiological processes [13].

In our previous study, *T. spiralis* Nd (TsNd) binding to normal mouse intestinal epithelial cells (IECs) were identified by screening a T7 phage display cDNA library from *T. spiralis* intestinal infective larvae (IIL) [14]. TsNd gene was also an up-regulated gene in IIL compared to muscle larvae (ML), which was identified by using suppression subtractive hybridization (SSH) and confirmed by real-time PCR [15]. The vaccination of mice with T7 Phage-displayed TsNd polypeptides (22 kDa) produced significant protective immunity against *T. spiralis* infection [16]. The results suggested that TsNd might play critical roles during the invasion of IECs by *T. spiralis*. However, the exact biological functions of TsNd are unknown.

In this study, the full-length cDNA sequence of TsNd gene (GenBank accession No. EU263318.1) encoding a 46 kDa protein from *T. spiralis* IIL was cloned and identified. The expression, immunolocalization of TsNd and the immune protection produced by the recombinant TsNd protein (rTsNd) was also investigated.

## Methods

### Ethics statement

This study was carried out in strict accordance with the National Guidelines for Experimental Animal Welfare (MOST of People’s Republic of China, 2006). All animal procedures reported herein were reviewed and approved by the Zhengzhou University Animal Care and Use Committee (Permission No. SYXK 2012–0009).

### Parasites and experimental animals

*T. spiralis* isolate (ISS534) was obtained from domestic pigs in Nanyang, Henan Province, China. Specific pathogen-free (SPF) male BALB/c mice aged 5 weeks were purchased from the Experimental Animal Center of Henan Province. All the animal experiments were approved by The Life Science Ethics Committee of Zhengzhou University.

### Collection of worms and preparation of crude and ES antigens

*T. spiralis* ML from infected mice at 42 days post-infection (dpi) were recovered by digestion of carcasses with 0.33% pepsin (1:31000; Sigma) and 1% HCl [17]. The IIL were collected from the mouse small intestines at 6 hpi, adult worms (AW) were isolated from the small intestines of infected mice at 3 and 7 dpi [18]. The newborn larvae (NBL) were collected from female adult worms cultured in RPMI-1640 medium containing 10% fetal bovine serum (FBS; Gibco) in 5% CO_2_ at 37°C for 24 h [19]. The crude antigens of ML, IIL, AW and NBL, and ES antigens of ML were prepared as previously described [20].

### Cloning, expression, and identification of TsNd

Total RNA was extracted from the IIL using Trizol (Invitrogen). TsNd gene was amplified by PCR, specific primers carrying *Bam*HI and pstI restriction enzyme sites (bold and italicized) (forward, 5’- TTA***GGATCC***ATGTTTTACTTGGTAACGCAGGCTAT -3’; reverse, 5’-TAT***CTGCAG***TTACCAAGTGTGTTGCAAAGCAATC -3’) were used. The purified PCR products were cloned into the pGEM-T vector (Promega, USA), and then were subsequently sub-cloned into the expression vector pMAL-c2X (New England Biolabs, USA). The expression of the rTsNd protein was induced with 0.5 mM IPTG for 4 h at 37°C. The rTsNd protein was expressed in supernatant and was purified by Amylose Pre-packed Column (NEB Ltd, China). The protein concentration of purified rTsNd was determined by Bradford method [21], analyzed by SDS-PAGE using a 5% acrylamide stacking gel and 12% acrylamide separating gel as described previously [22]. N-terminal maltose-binding protein (MBP) tag was expressed and purified by the above-mentioned method.

### Sequence analysis of the TsNd gene

The full-length cDNA sequence of TsNd gene was analyzed, the conceptual translation of cDNA into amino acid sequence was performed using the open reading frame (ORF) finder program from the NCBI [23]. Amino acid sequence of TsNd was submitted to http://www.expasy.org/tools/protparam.html and its physical and chemical properties were predicted. The signal peptide was predicted by SignalP 4.1 Server [24]. The domain analysis of TsNd was performed using Conserved Domain Database of NCBI. Transmembrane domain was predicted through http://www.cbs.dtu.dk/services/TMHMM/, and subcellular localization was predicted using http://psort.nibb.ac.jp/form2.html. Sequence alignments were analyzed with protein–protein basic local alignment search tool (BLASTp) and BLAST2 sequences using default parameters and with Clustal W [25].

### Phylogenetic analysis of TsNd

The Nd amino sequences of *T. spiralis* and other organisms used in this study were obtained from GenBank. The amino acid sequences data were aligned using Clustal X; then, molecular evolutionary tree was constructed by MEGA 6.0 [26]. Phylogenies were estimated under the maximum parsimony (MP) method [27,28].

### Immunization of mice

Eighty BALB/c mice were divided into four groups of 20 animals each. The vaccine group of mice was immunized with rTsNd. Each mouse was subcutaneously vaccinated with 20 μg of rTsNd emulsified with complete Freund’s adjuvant, followed by two boosts with the same amount of protein emulsified with incomplete Freund’s adjuvant at 10 day intervals [29]. The control groups of mice were injected only with MBP tag protein, adjuvant or PBS using the same immunization schedule. About 50 μl of tail blood of immunized mice were collected at 0, 10, 20, and 30 days post-immunization.

### Antibody determination

The levels of the specific total IgG, IgG1 and IgG2a antibodies to rTsNd in serum of immunized mice were determined by ELISA with rTsNd as described previously [30]. Briefly, microtiter plates (Nunc) were coated with rTsNd (1 μg/ml) in coating buffer overnight at 4°C, and blocked with 200 μl of PBS-0.1% Tween 20 (PBST) containing 5% skimmed milk. Then, 100 μl of immune serum with serial dilutions were added to each well and incubated at 37°C for 1 h. HRP-conjugated goat anti-mouse IgG antibodies (1:5000; Southern Biotechnology, USA) were added and incubated at 37°C for 1 h. The reactions were detected by addition of the substrate o-phenylenediamine dihydrochloride (OPD; Sigma) plus H_2_O_2_ and stopped with 50 μl/well of 2 M H_2_SO_4_. Absorbance at 490 nm was measured with a microplate reader (TECAN, Austria). All samples were run in duplicate.

### Western blot analysis

Protein samples including crude antigens of ML, IIL, AW and NBL, ES antigens of ML, and rTsNd were separated by SDS-PAGE using a 10% acrylamide separating gel and then transferred onto nitrocellulose membranes (Millipore, USA) using a trans-blot SD transfer cell (Bio-Rad, USA) [31,32]. The membranes were cut into strips, blocked with 5% skimmed milk in Tris-Buffered Saline with Tween-20 (TBST) at 37°C for 1 h, and incubated at 37°C for 1 h with 1:100 dilutions of different mouse sera. After being washed, the strips were incubated at 37°C for 1 h with HRP-conjugated goat anti-mouse IgG (1:5000 dilution), and finally with 3, 3’-diaminobenzidine tetrahydrochloride (DAB; Sigma).

### RT-PCR

Total RNA was extracted from IIL, AW, NBL and ML of *T. spiralis* using Trizol reagent (Invitrogen), respectively. RT-PCR was performed as previously described [29]. The housekeeping gene GAPDH (glyceraldehyde-3-phosphate dehydrogenase, GenBank accession No. AF452239) of *Trichinella* was also amplified as a positive control, the primers were designed as follows: forward, 5’-TTAATGTCGTGGCTGTGAAT-3’, and reverse, 5’-CCAGTAGAAGCAGGGATGAT-3’. As a negative control, PBS was used as template in all PCRs.

### Immunofluorescent test (IFT)

Anti-rTsNd serum was collected from the mice immunized with rTsNd at 10 days after the last immunization, the specific IgG antibody titer was 1:10^6^ assayed by ELISA. The ML at 42 dpi, IIL at 6 hpi, and AW 3 dpi were fixed in 4% paraformaldehyde and embedded in paraffin. Microtome-cut 2 μm sections were placed on slides, deparaffinized in xylene and rehydrated. The whole parasites and sections were blocked with 5% normal goat serum in PBS, incubated in a moist chamber at 37°C for 1 h with a 1:10 dilution of anti-rTsNd serum, infection serum or normal mouse serum. After washing three times in PBS, the whole parasites and sections were incubated with a 1:100 dilution of FITC-labeled goat anti-mouse IgG (Santa Cruz, USA). After being washed five times with PBS, the whole parasites were re-dyed with 0.01% Evans blue and the sections were not re-dyed with Evans blue, and examined under a fluorescent microscope (Olympus, Japan) [16].

### Challenge infection

Ten days after the last immunization, four groups of mice (20 mice for each group) were challenged orally with 300 ML for per mouse. Ten mice from each group were euthanized at 3 dpi and the numbers of intestinal adult worms were counted [33]. The muscle larvae were examined from the remaining 10 mice from each group at 35 dpi by artificial digestion [34]. The protective immunity was calculated as the worm reduction rate of adults and larvae per gram (LPG) of muscle recovered from the immunized groups versus those from the control groups [35].

### Statistical analysis

All of the statistical analyses of the data were performed using SPSS for Windows, version 17.0 (SPSS Inc., Chicago, IL). The data of AW and ML recovery, and absorbance were expressed as the mean value ± standard deviation, and the intra- and intergroup differences were analyzed by using one-way ANOVA or Student’s t test. *P* < 0.05 was considered as statistically significant.

## Results

### Cloning and identification of the cDNA encoding TsNd protein

**Figure 1:**
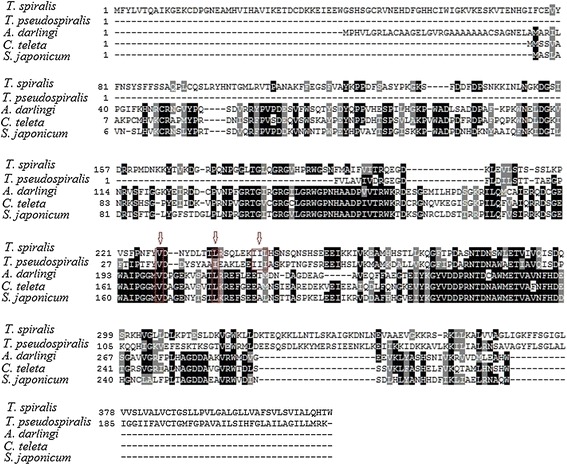
**Amino acid sequence alignment of Nudix hydrolase from** ***Trichinella spiralis*** **(ABY60748.1) with** ***Schistosoma japonicum*** **(CAX75016.1),** ***Anopheles darlingi*** **(ETN60541.1),** ***Trichinella pseudospiralis*** **(ABL09502.1), and** ***Capitella teleta*** **(ELT87584.1) using Clustal W.** Sequences were aligned using CLUSTALW and prepared for display using BOXSHADE. Residues identical to TsNd are shaded black, and conservative substitutions are shaded grey. Arrow shows the putative metal binding site/catalytic sites.

**Figure 2:**
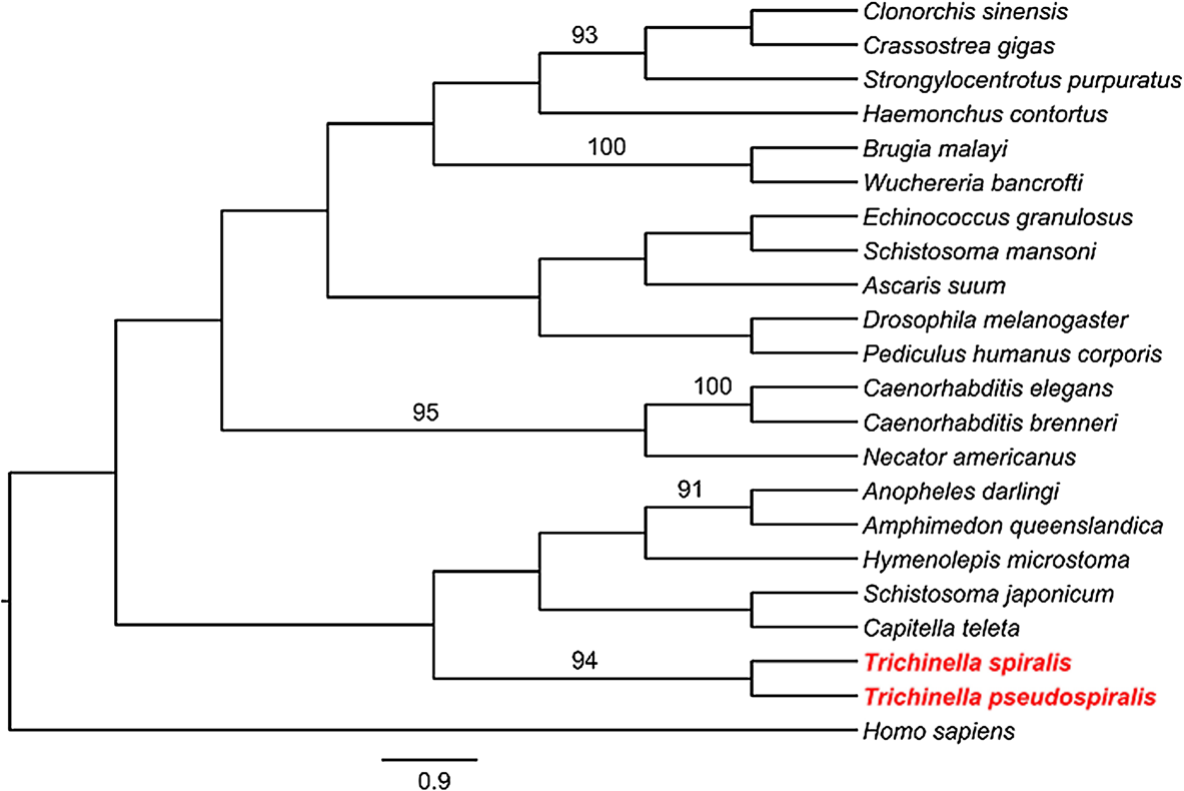
**Phylogenetic tree of Nudix hydrolase from 22 organisms using the Maximum parsimony method and plotted with MEGA.** The GenBank accession number of each Nudix hydrolase is: *T. spiralis* (ABY60748.1), *C. elegans* (O45830.1), *Haemonchus contortus* (CDJ97233.1), *Necator americanus* (ETN70016.1), *Ascaris suum* (ADY47382.1), *Brugia malayi* (EDP34320.1), *Trichinella pseudospiralis* (ABL09502.1), *Echinococcus granulosus* (CDJ23656.1), *Clonorchis sinensis* (GAA49418.1), *Crassostrea gigas* (EKC30073.1), *Anopheles darlingi* (ETN61723.1), *Homo sapiens* (CAD01139.1), *Schistosoma japonicum* (AAW26860), *Pediculus humanus corporis* (EEB16642.1), *Schistosoma mansoni* (CCD60247.1), *Hymenolepis microstoma* (CDJ09496), *Wuchereria bancrofti* (EJW73682), *Caenorhabditis brenneri* (EGT47426), *Drosophila melanogaster* (CAI10730.1), *Capitella teleta* (ELT97831), *Amphimedon queenslandica* (XP_003389450), *Strongylocentrotus purpuratus* (XP_003729509.1), *Pediculus humanus corporis* (EEB16642.1). Bootstrap values which are higher than 90 are indicated on branches. The tree was rooted using *Homo sapiens*.

The results of cloning and sequencing showed that the cDNA of TsNd was about 1248 bp. The start codon “ATG” is located at 111–113 bp, and the stop codon located at 1356–1358 bp. The predicted open reading frame (ORF) of TsNd encodes 415 amino acids with a molecular weight of 46 kDa and an isoelectric point (pI) of 8.85. SignalP 4.1 Server predicted there were no signal peptides. Conserved Domain analysis revealed there are two domains of TsNd, Nudix motif and YhfT. Nd has a nudix motif located at 226–244 aa which is the active sites of binding metal irons. YhfT was located at 365–415 aa and its function was unknown. Prediction of transmembrane domain of TsNd suggested that TsNd had a trans-membrane domain (between positions 393 and 411 aa). The prediction results of subcellular localization showed that TsNd might be located in endoplasmic reticulum (44.4%), mitochondria (22.2%), plasma membrane (22.2 %), and vesicles of secretory system (11.1%). Results of similarity analysis showed that the deduced amino acid sequence of TsNd had high homology with the Nd from *T. pseudospiralis* (Figure 1). The phylogenetic analysis of TsNd with the Nd of other organisms was shown in Figure 2. Based on the phylogenetic analysis of Nd, *T. spiralis* has the closest evolutionary status with *T. pseudospiralis.*

### Expression of recombinant TsNd protein

**Figure 3:**
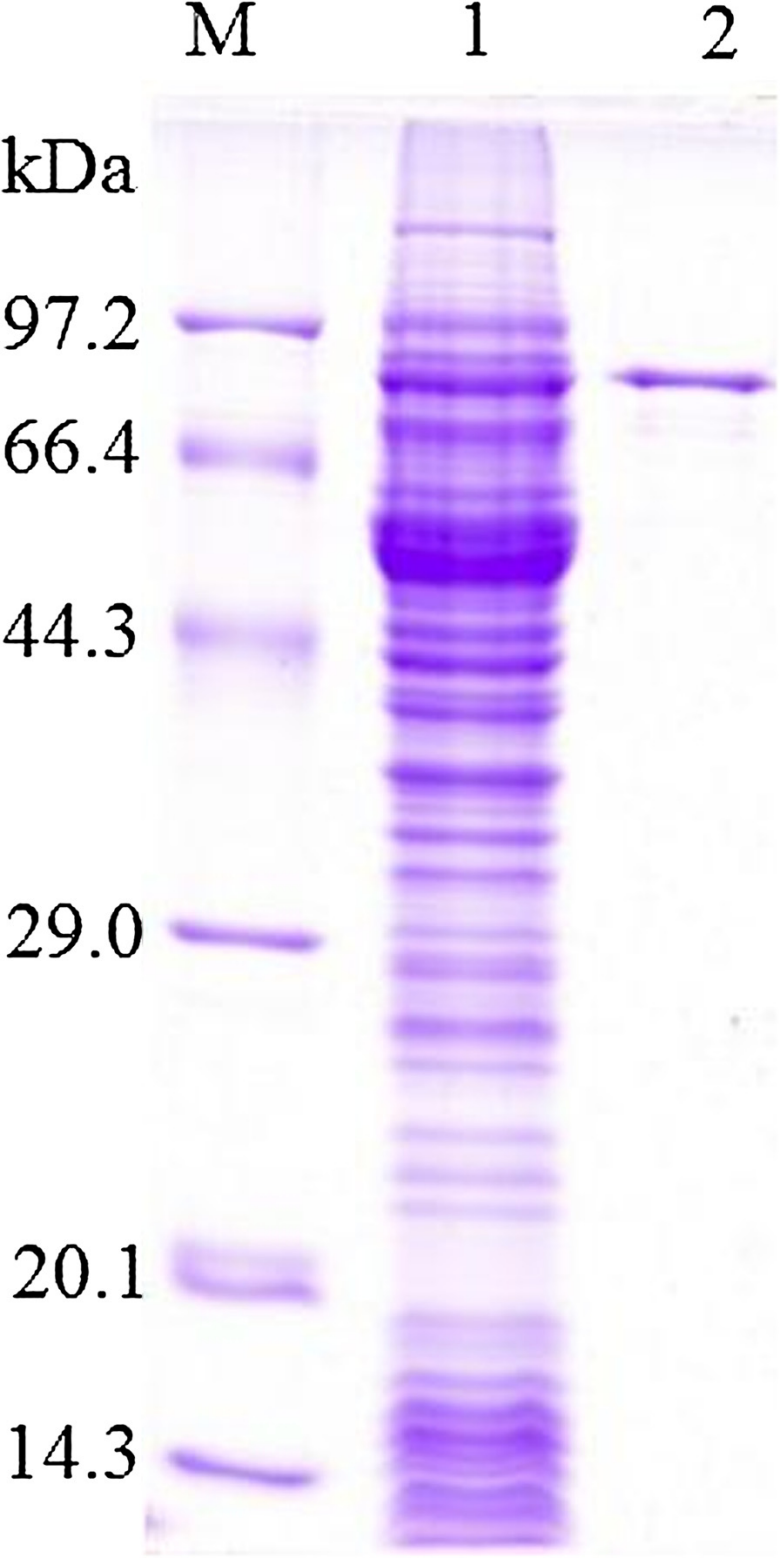
**SDS-PAGE analysis of recombinant TsNd protein.** M: protein molecular weight marker; 1: the lysis of the induced recombinant bacteria; 2: rTsNd purified by affinity chromatography using amylose resin.

After induction with 0.5 mM IPTG, BL21 (DE3) bacteria harboring pMAL-c2X-TsNd expressed a band of 89 kDa fusion protein. On SDS-PAGE analysis, the molecular size of the rTsNd protein was consistent with the predicted combined size of the protein encoded by the cDNA clone (46 kDa) and MBP tag from the vector (43 kDa) (Figure 3).

### Western blot analysis of the recombinant TsNd protein

**Figure 4:**
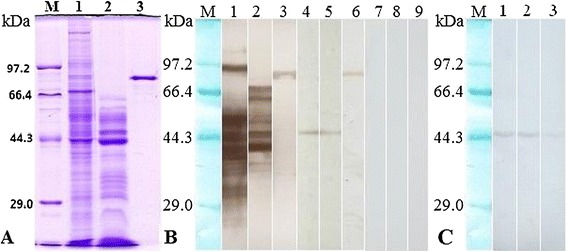
**Identification of recombinant TsNd protein. (A)** The SDS-PAGE analysis of *T. spiralis* ML crude antigens (lane 1), ES antigens (lane 2), and rTsNd. **(B)** Western blot analysis of rTsNd antigenicity. The *T. spiralis* ML crude proteins (lane 1), ES proteins (lane 2), and rTsNd (lane 3) were recognized by sera of mice infected with *T. spiralis* at 42 dpi. The native TsNd protein in crude antigens (lane 4), ES antigens (lane 5), and rTsNd (lane 6) were recognized by anti-rTsNd serum. The *T. spiralis* crude proteins (lane 7), ES proteins (lane 8), and rTsNd (lane 9) were not recognized by sera of normal mice. **(C)** Western blot analysis of the crude antigens of *T. spiralis* different developmental stages by using anti-rTsNd serum. The native TsNd protein in crude antigens of *T. spiralis* IIL at 6 hpi (lane 1), AW at 3 dpi (lane 2), and NBL (lane 3) were recognized by anti-rTsNd serum.

Western blot analysis showed that the rTsNd was recognized by anti-rTsNd serum and serum of *T. spiralis-*infected mice (Figure 4). Anti-rTsNd serum recognized the native TsNd protein with a molecular weight of 46 kDa in crude antigens of ML, IIL, AW and NBL, and ES antigens of ML, indicating that TsNd is one component of both the crude and ES proteins from *T. spiralis*.

### RT-PCR analysis of TsNd gene transcription

**Figure 5:**
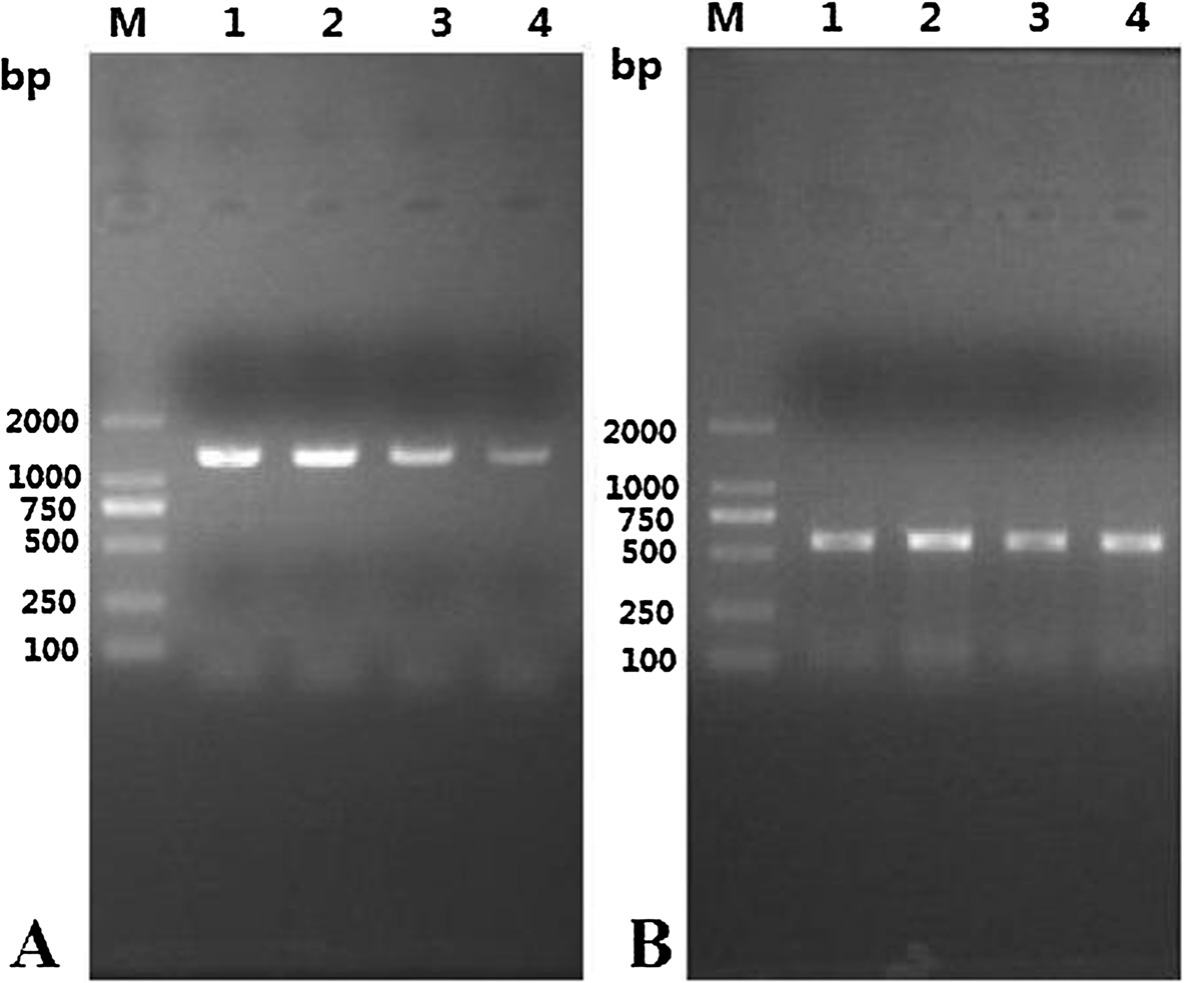
**RT-PCR analysis of TsNd gene transcript at different stages of** ***T. spiralis*** **.** RT-PCR detection of the mRNA transcription for TsNd gene **(A)** and GAPDH gene **(B)** at different developmental stages of *T. spiralis*. M: DNA marker; Lane 1: ML at 42 dpi; Lane 2: IIL at 6 hpi; Lane 3: AW at 3 dpi; Lane 4: NBL.

The mRNA transcript (1248 bp) for the TsNd gene was detected at different developmental stages of *T. spiralis*. Additionally, the transcription level of TsNd gene in ML at 42 dpi and IIL at 6 hpi was higher than that in AW at 3 dpi and NBL (Figure 5A). Furthermore, the primers for a standard gene (GAPDH) generated the expected size (570 bp) band in all of the samples (Figure 5B).

### Expression and immunolocalization of TsNd at different developmental stages

**Figure 6:**
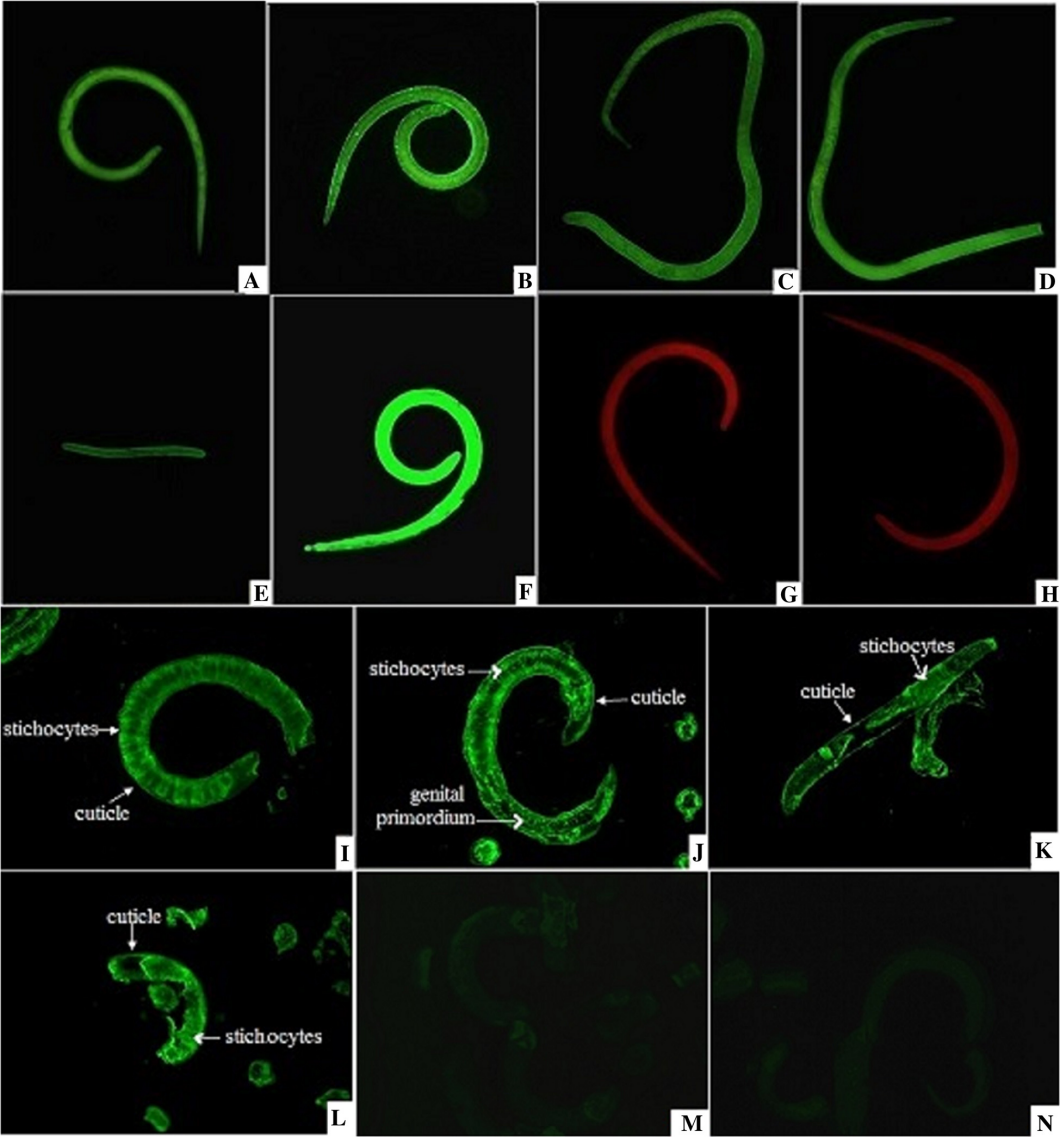
**Expression of TsNd at different developmental stages and immunolocalization in** ***T. spiralis*** **. A-H:** The results of IFT with whole parasite of *T. spiralis* different stages reacted with anti-rTsNd serum. The notable green fluorescent staining is found on the surface and in the body of ML at 42 dpi **(A)**, IIL at 6 hpi **(B)**, female AW at 3 dpi **(C)**, male AW at 3 dpi **(D)** and NBL **(E)**.The ML with mouse infection serum **(F)** as a positive control; ML did not show recognition by normal mouse serum **(G)** and PBS **(H)** as a negative control. **I–N**: The results of IFT with the sections of whole worms (ML at 42 dpi, IIL at 6 hpi and AW 3 dpi) reacted with anti-rTsNd serum. The immunostaning is seen at the cuticle, stichocytes and genital primordium of ML **(I)**, IIL **(J)** and AW **(K)**. The ML reacted with mouse infection serum **(L)** as a positive control; ML did not show recognition by normal mouse serum **(M)** and PBS **(N)** as a negative control.

The results of IFT with the whole parasite showed that the intense green fluorescent staining using anti-rTsNd serum was found at all different developmental stages of *T. spiralis* (e.g., ML at 42 dpi, IIL at 6 hpi, AW at 3 dpi and NBL), and the stronger staining was observed in IIL (Figure 6). When the sections of the parasite were incubated with anti-rTsNd serum, positive green fluorescent staining was found at the cuticle, stichocytes and reproductive organs of ML at 42 dpi, IIL at 6 hpi, and AW at 3 dpi.

### Humoral immune responses induced by vaccination with rTsNd

**Figure 7:**
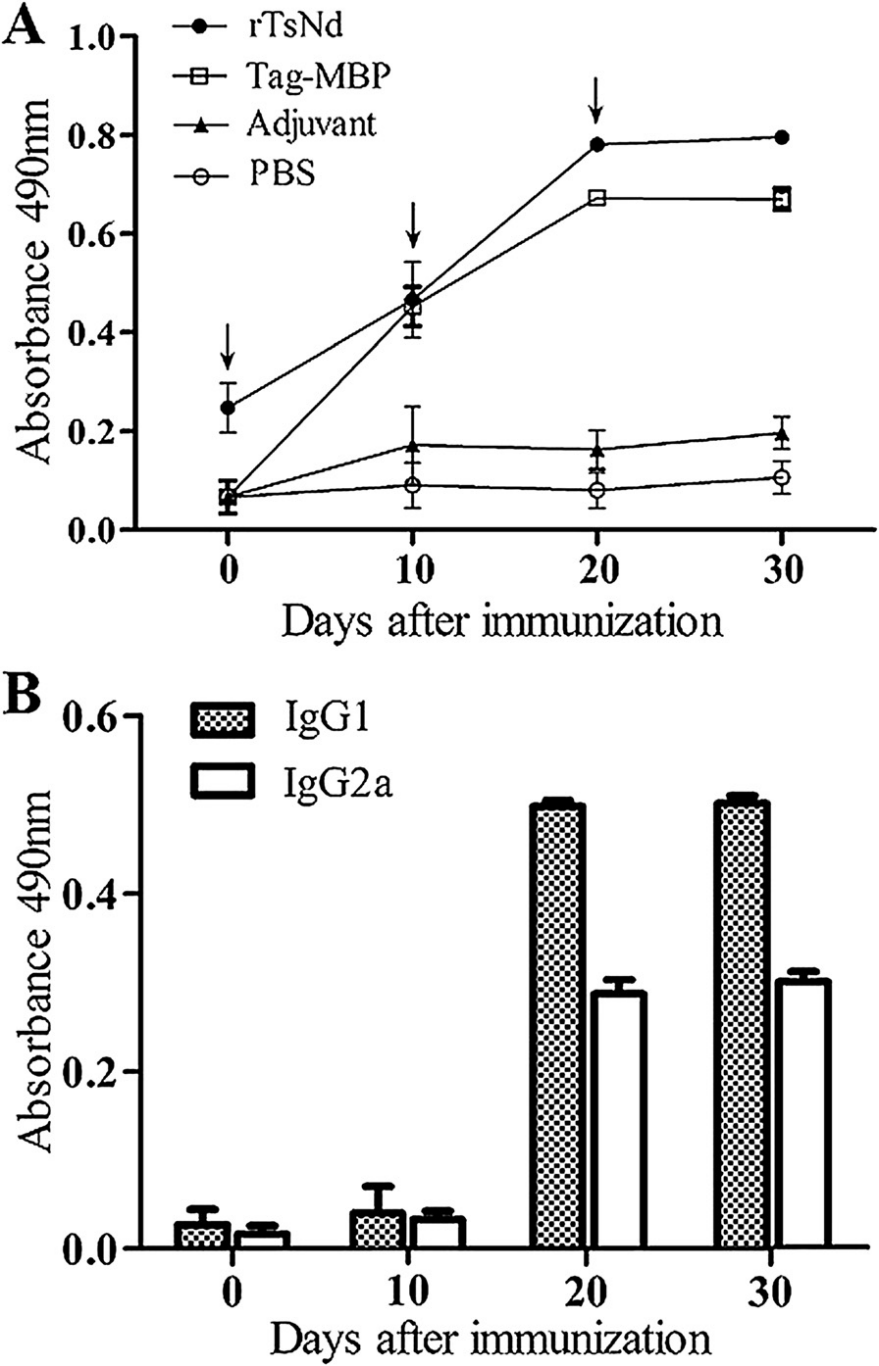
**Analysis of antibody responses in mice. (A)** Anti-rTsNd IgG levels in the sera of immunized mice or control (MBP tag, adjuvant or PBS) mice were measured by ELISA. **(B)** The IgG subclass (IgG1 and IgG2a) responses in immunized mice were detected at different time point post vaccination. The OD values shown for each group are the mean ± SD of the antibody levels. The immunization time points are marked with an arrow (↓).

Anti-rTsNd IgG levels in mice immunized with rTsNd were greatly increased following the first and second immunization (Figure 7). However, the mice vaccinated with MBP Tag had also high levels of IgG antibodies, which might be related with the rTsNd contained the MBP tag. But none of the mice vaccinated with adjuvant or PBS show significantly detectable rTsNd-specific responses. The results of IgG subclass antibody assay showed that after the second and third immunization, the levels of IgG1 were more significantly higher than that of IgG2a (t_20d_ = 39.485, t_30d_ = 45.422, *P* < 0.01).

### Immune protection of recombinant TsNd against challenge infection

**Figure 8:**
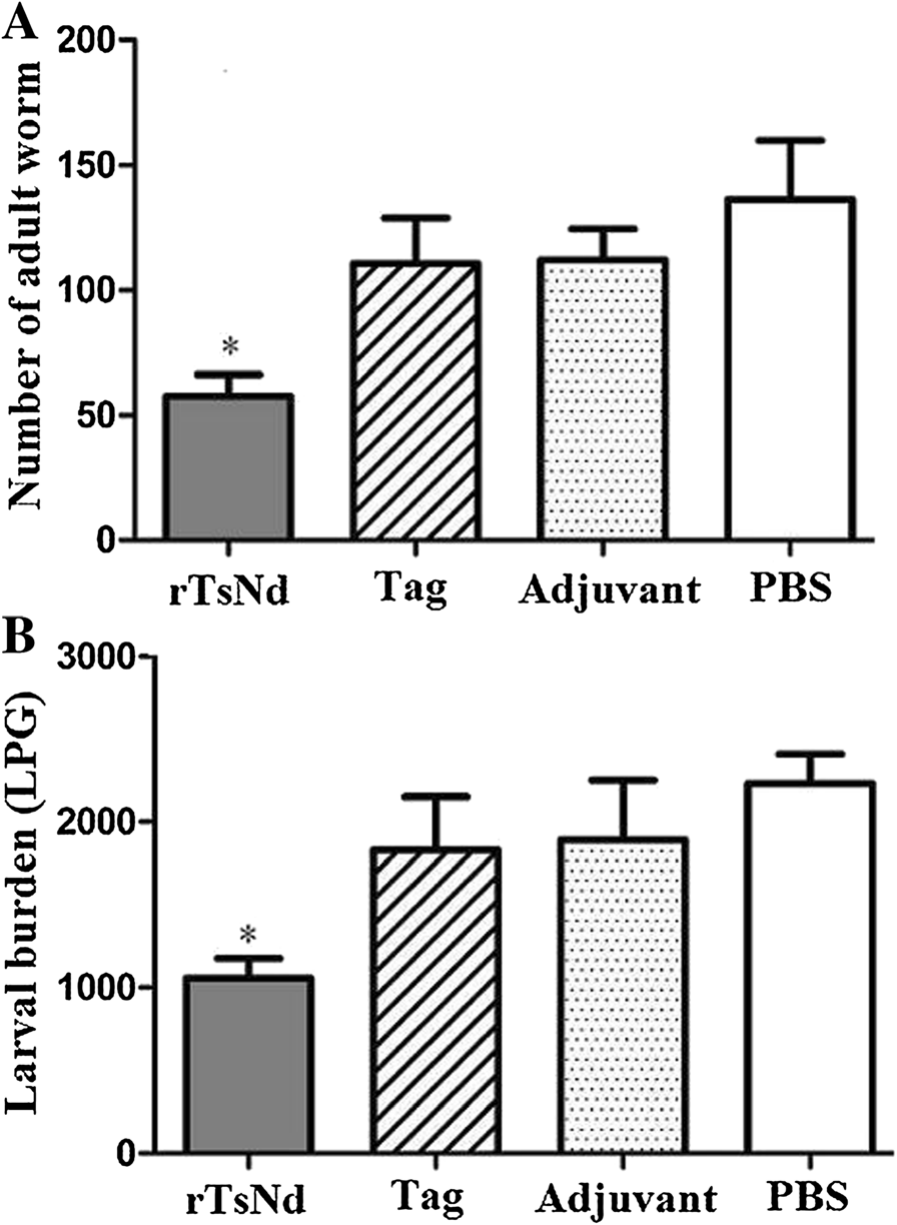
**The number of adult worms (A) and larvae per gram (LPG) of muscles (B) recovered from vaccinated mice after a challenge infection with 300** ***T. spiralis*** **larvae.** Results are presented as the arithmetic mean ± standard deviation (SD) of ten mice each group. Asterisks (*) indicate statistically significant differences (*P* < 0.05) in worm recovery of the immunized group compared to three control groups.

Protective immunity against *T. spiralis* infection induced by rTsNd was observed in immunized BALB/c mice. After the challenge infection with *T. spiralis* ML, the mice immunized with rTsNd displayed a 57.7% reduction in intestinal adult worms (Figure 8A) and a 56.9% reduction in the muscle larval burden (Figure 8B) compared with the PBS control groups. Further, the number of adults and larvae recovered from the immunized group was significantly lower than that from the tag protein, adjuvant and PBS control groups (F_adults_ = 4.502, F_larvae_ =11.628, *P* < 0.01).

## Discussion

Sequence analysis of TsNd gene showed that there was an open reading frame of 415 aa, and a conserved domain of the Nd superfamily which is supposed to the mainly biological function domain. The amino acid sequence of TsNd showed high homology with Nd of *T. pseudospiralis.*

*E. coli* expression system has been considered as the most commonly used for gene engineering to produce proteins. In this study, the TsNd gene encoding a 46 kDa protein from *T. spiralis* was successfully cloned and expressed in *E. coli* using the MBP fusion-based pMAL-c2X vector, and the produced rTsNd was soluble. After purification, rTsNd was used as an immunogen to produce anti-rTsNd antibodies, and the resulting anti-rTsNd serum was used to define some native characteristics of TsNd [29]. Our results showed that the immunized mice produced strong specific antibodies to rTsNd and the IgG antibody titer was 1:10^6^. Western blot analysis showed that anti-rTsNd serum obviously recognized the native TsNd protein in crude antigens of IIL, AW and NBL, and ES antigens of ML, indicating that TsNd is one component of both the crude and ES proteins from *T. spiralis*.

The characteristics of TsNd were identified at transcription and expression levels of TsNd using RT-PCR and IFT. As shown in Figure 5, the results of RT-PCR showed that the TsNd was transcribed during all *T. spiralis* developmental stages (ML, IIL, AW and NBL), but the transcription level of TsNd in ML and IIL was higher than that in AW and NBL. The IFT revealed positive immunostainings were widely distributed in the body (cuticle, stichocytes and reproductive organs) of the whole or sections of parasites (ML, IIL, AW and NBL) incubated with anti-rTsNd serum; the green fluorescent staining in IIL is more obvious than that in other stages. The results indicated that TsNd gene was transcribed and expressed during all the developmental stages of *T. spiralis*, and has a high level of transcription and expression in the IIL stage, possibly because TsNd was an invasion-related protein of IIL [15]. The prediction results of subcellular localization showed that TsNd might be located mainly in endoplasmic reticulum, mitochondria and plasma membrane. Nd is a family of proteins that contain the characteristic sequence of Nudix box. They catalyze the hydrolysis of a variety of nucleoside diphosphate derivatives [36]. Substrates of Nudix enzymes are either potentially toxic, deleterious compounds, such as ADP-ribose (ADPR) and 8-oxo-GTP, or important cell signaling molecules, regulators, or metabolic intermediates such as CoA, NADH, and dATP, *etc*. [13]. These “housecleaning” activities appear essential for the organism [37]. Another study showed that *E. coli* NudH was contributed to invasion of human brain microvascular endothelial cell by *E. coli* [38]. These results suggested that TsNd might be an indispensable protein and played an important role in the life cycle of *T. spiralis* and larval invasion of IECs.

After the challenge infection with *T. spiralis* ML, the mice immunized with rTsNd displayed a 57.7% reduction in adult worms and a 56.9% reduction in the muscle larvae. The results showed that the vaccination of mice with rTsNd induced a partial protective immunity. The adult worm reduction observed in this study is similar with those from the previous reports, but the muscle larval reduction is lower [16]. The difference might be related with the different adjuvant and buffer used in control groups. Since the rTsNd is a MBP-TsNd fusion protein, the mice vaccinated with MBP Tag could also produce anti-MBP antibodies. Anti-MBP tag antibodies in serum of immunized mice were detected by rTsNd, but they have no killing effect on *T. spiralis*. Hence, the vaccination of mice with MBP tag induced the high levels of IgG antibodies, but conferred no immune protection against *T. spiralis* infection. The increased IgG1 isotype demonstrated that the Th2-predominant immune responses had been successfully induced by rTsNd. It is suggested that the immune protection conferred by the immunization with rTsNd might be due to production of anti-rTsNd antibodies that neutralise TsNd enzyme activity [39]. The immune protection also might be related to the blockage of the larval invasion of IECs by specific antibodies due to the formation of larval cephalic immune complexes [40,41]. However, the exact biological functions and immune protection mechanism of TsNd have not been fully clarified. Further experiments *in vitro* and *in vivo* are needed to determine the roles of TsNd in the process of *T. spiralis* invasion and development.

## Conclusions

The present study showed that transcription and expression of TsNd were observed at all the developmental stages of *T. spiralis*, and located in the cuticle, stichocytes and reproductive organs of the parasite. The vaccination of mice with rTsNd induced the Th2-predominant immune responses and a partial protective immunity against *T. spiralis* infection. TsNd could be considered as a novel candidate vaccine antigen against trichinellosis.
